# Scaphocapitate Fracture Syndrome in an Adolescent: A Case Report, Diagnosis, and Surgical Treatment

**DOI:** 10.7759/cureus.96213

**Published:** 2025-11-06

**Authors:** Maria Sakellariou, Myrto Skouteli, Emmanouil Apergis, Yvonne-Mary Papamerkouriou

**Affiliations:** 1 Orthopaedics, Panagiotis & Aglaia Kyriakou Children's Hospital, Athens, GRC; 2 Orthopaedics, Korgialenio-Benakio Hellenic Red Cross General Hospital, Athens, GRC

**Keywords:** 180 degree rotation, adolescent, fenton syndrome, hand surgery, scaphocapitate fracture syndrome

## Abstract

Scaphocapitate fracture syndrome, or Fenton syndrome, is a rare condition characterized by fractures of the scaphoid and capitate bones, often with a 180-degree rotation of the proximal capitate, and is frequently underdiagnosed due to its rarity and inconclusive initial radiographic findings. We report the case of a 15-year-old boy who sustained a high-energy wrist injury after a three-meter fall. Initial X-ray raised suspicion of a scaphocapitate fracture syndrome; therefore, the patient was referred for a CT scan. He returned with the results two months later, which confirmed scaphocapitate fracture syndrome. Surgical management involved open reduction and internal fixation of the scaphoid and capitate fractures, repair of the iatrogenic lunate fracture, and external fixation for stability. Postoperative recovery was satisfactory with complete fracture healing, no avascular necrosis, and minimal functional limitations at the five-year follow-up with a Modified Mayo Wrist Score of 100 out of 100 points. This case highlights the importance of high clinical suspicion, advanced imaging for accurate diagnosis, and anatomical surgical fixation to ensure alignment and favorable long-term outcomes.

## Introduction

The scaphocapitate fracture syndrome, also known as Fenton syndrome, is a rare clinical condition with an estimated incidence of approximately 0.6% of the fractures of the carpal bones and is often misdiagnosed. The term scaphocapitate fracture syndrome refers to the simultaneous occurrence of fractures in the capitate and scaphoid bones, accompanied by a 90- or 180-degree rotation of the proximal capitate bone. The degree of rotation is a prognostic factor for each case [1-3]. This clinical entity was first described by Fenton and Rose in 1950 [4]. It typically manifests following a traumatic wrist injury, particularly in young adults [4,5], with its description being rare in the literature. Scaphocapitate syndrome often goes undiagnosed due to the presence of multiple coexistent injuries and the low incidence of fractures of the capitate bone [2,6]. However, advancements in high-resolution micro-computed tomography (mCT) imaging technology have revealed that avascular necrosis, following capitate bone fracture, is rare, attributed to its distinct dorsal and volar vascular supplies. Consequently, the capitate bone emerges as a potential candidate for delayed fixation [7]. We present the case of a young boy with a history of a fall, who sustained a scaphocapitate fracture.

## Case presentation

**Figure 1:**
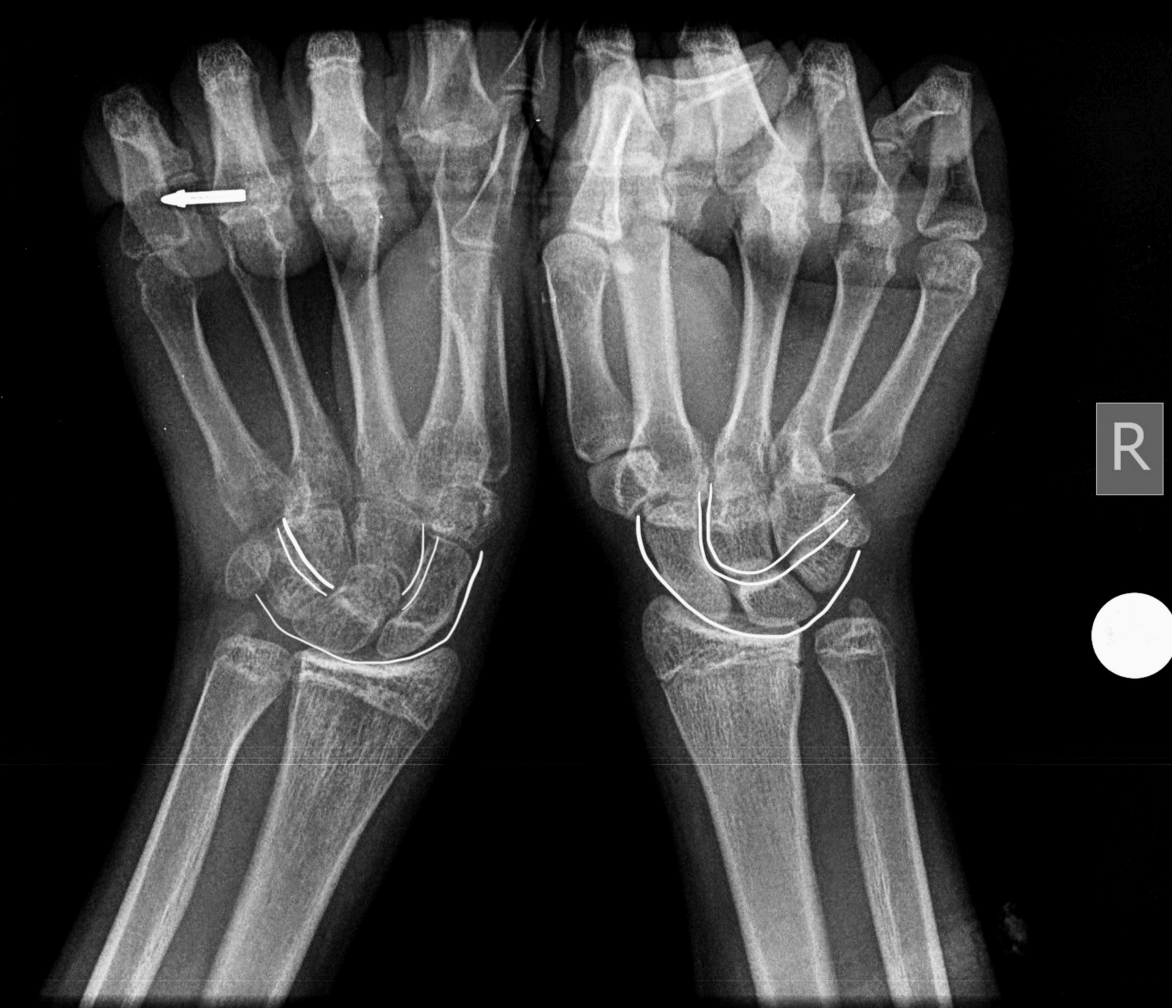
: X-rays of both hands with Gilula’s lines drawn (proximal, medial, distal), showing that medial and distal Gilula’s lines are interrupted in the left hand.

**Figure 2:**
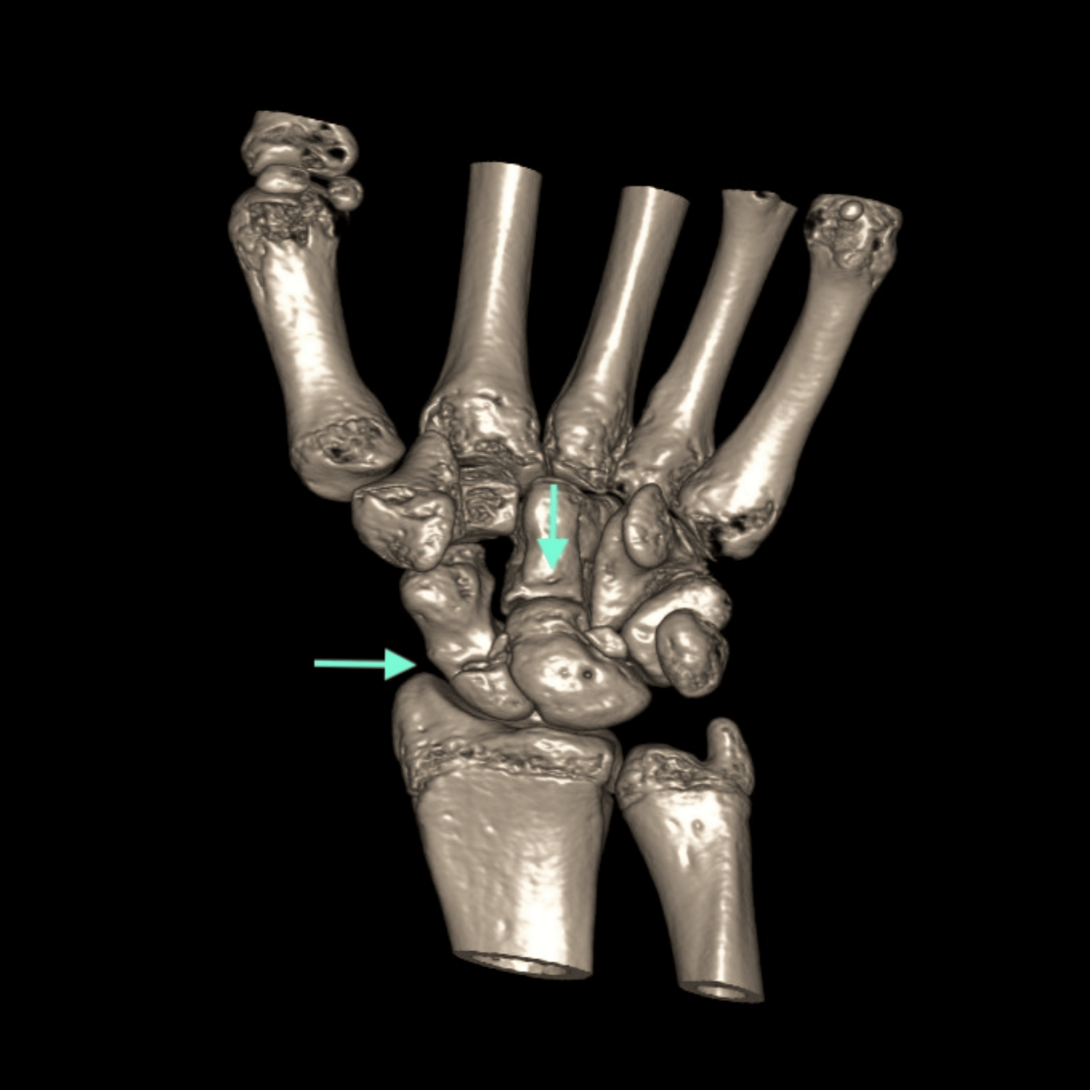
A 3D CT imaging depiction of the scaphoid fracture and the capitate fracture with rotation of the proximal pole.

**Figure 3:**
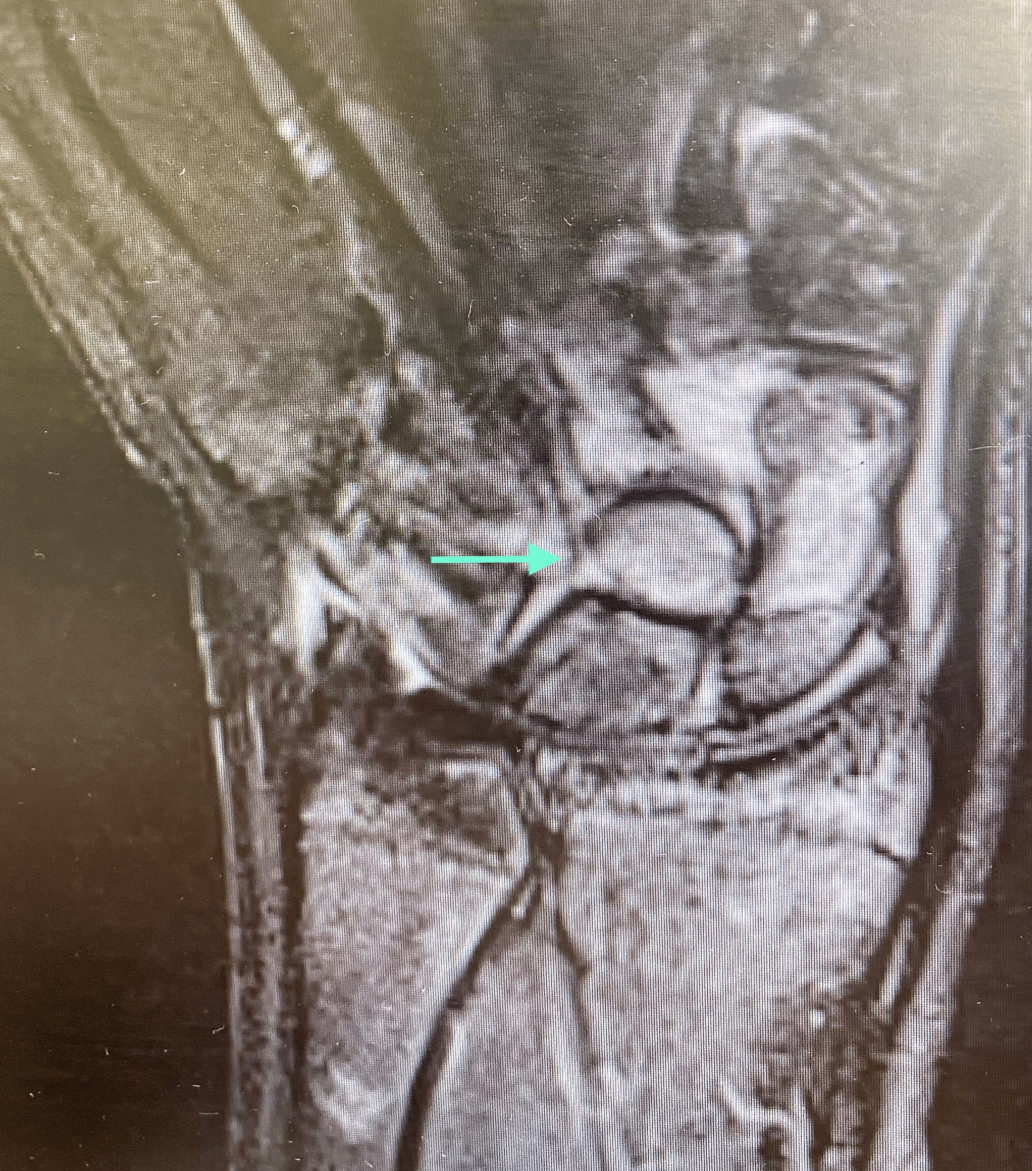
MRI imaging depicting a 180 rotation of capitate bone and no apparent avascular necrosis.

A 15-year-old left-hand-dominant boy with no known comorbidities sustained a high-energy injury after falling from a height of three meters on an outstretched left arm with the wrist in hyperextension. He presented the same day to the emergency department with severe pain and swelling. Physical examination revealed a restricted range of motion, with intact skin and a normal neurovascular assessment. Radiographic imaging with anteroposterior, lateral, and oblique views showed a fracture of the proximal pole of the scaphoid and an abnormal contour of the capitate bone, raising high suspicion of a fracture. In our case, the interruption of the medial and distal Gilula’s lines was evident on X-ray (Figure 1). Okoro et al. have recommended the use of Gilula's lines for the accurate assessment of carpal bone injuries, which are drawn proximally, medially, and distally across the carpal rows [8]. The injury was treated initially by cast immobilization, and a CT scan was ordered, but, unfortunately, the patient delayed returning for follow-up. He returned two months later with persistent pain. Diagnosis of the syndrome was confirmed by CT scan results showing both scaphoid and capitate fractures (Figure 2). An MRI was performed to rule out avascular necrosis, which confirmed the fracture of the capitate head with a 180-degree rotation of the proximal pole (Figure 3), as well as the scaphoid fracture. No avascular necrosis was apparent, and therefore surgery was decided, by which time, three months had passed post injury.

**Figure 4:**
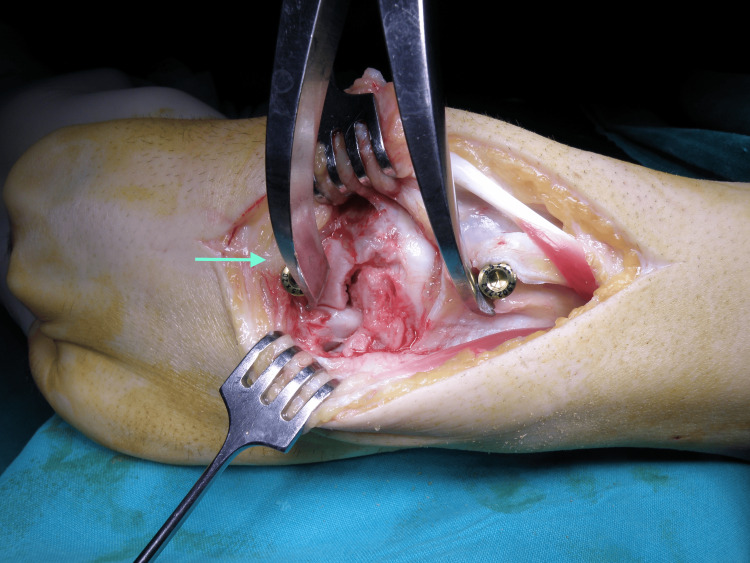
The technique of using the laminar spreader.

The operation was performed under general anesthesia, tourniquet control above the elbow, and fluoroscopy. A dorsal approach to the wrist through the fourth dorsal compartment was used. The scaphoid and capitate fractures were managed with open reduction and internal fixation. The laminar spreader technique was employed at the start of the procedure, using dorsally and distally placed screws in the capitate and radius to serve as anchors for distraction of the lunate space (Figure 4).

**Figure 5:**
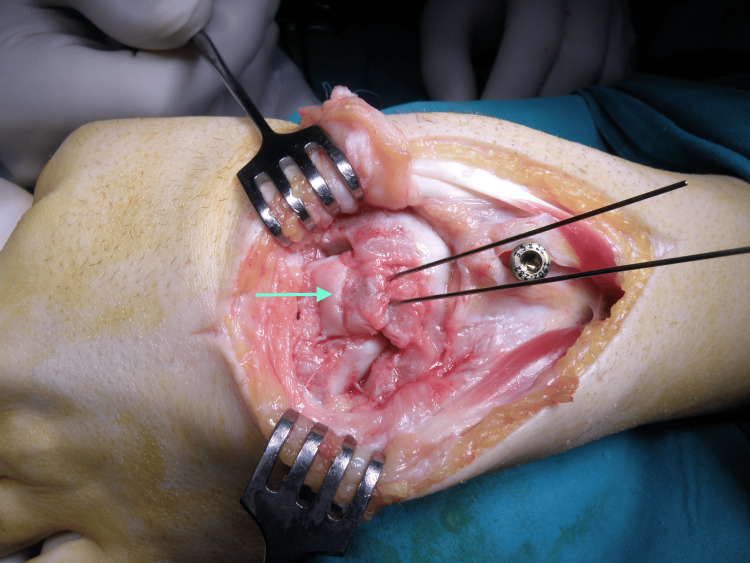
Stabilised capitate fracture with two K-wires.

The next step in the surgical procedure involved a near-atraumatic technique to achieve a 180-degree de-rotation of the proximal capitate fracture. This process facilitated optimal fixation of the distal capitate articular surface and addressed the more challenging proximal fixation. The capitate fracture was stabilised with two K-wires (Figure 5) and treated using two cannulated, headless compression screws (1.7 mm) inserted into the head of the capitate to facilitate compression. The scaphoid fracture was reduced and stabilized with a cannulated compression screw (3.0 mm).

**Figure 6:**
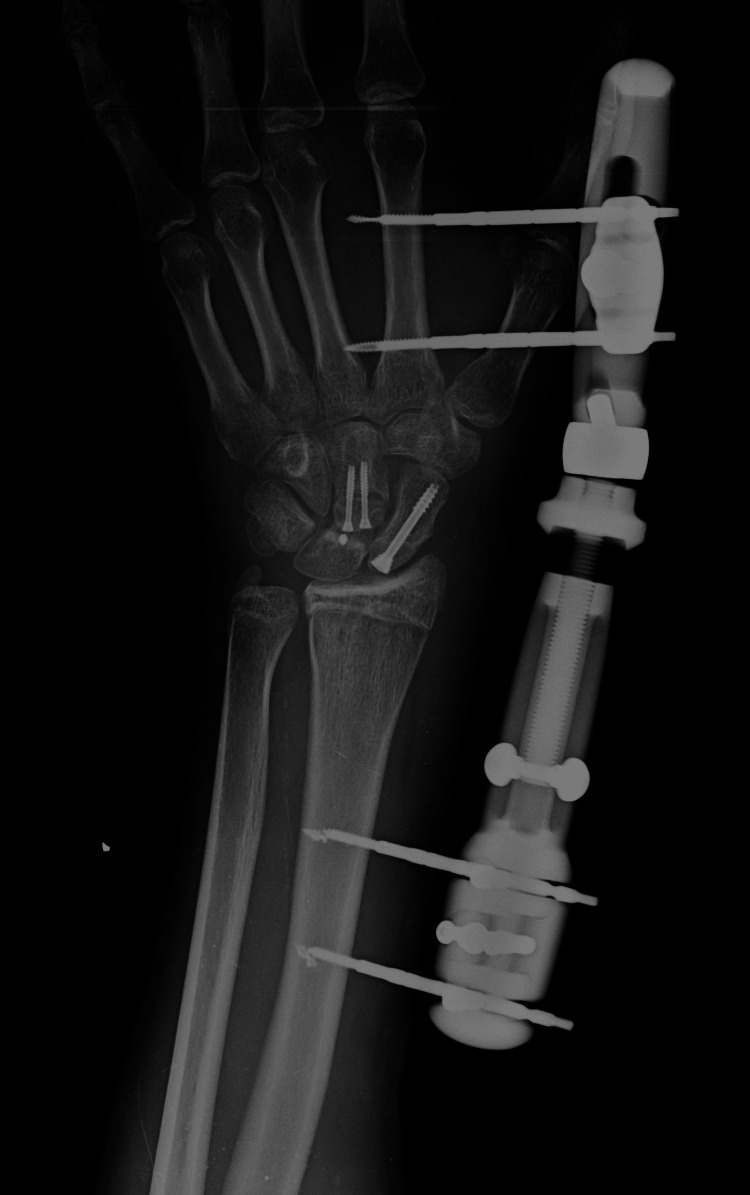
Postoperative X-ray showing internal fixation of carpal bones with screws and external fixation placement for alignment.

During the procedure, an avulsion of the dorsal pole of the lunate bone was inadvertently induced. This was subsequently stabilized using a bone anchor, and the fracture was secured with bone sutures that extended into the synovial bursa. At the conclusion of the procedure, external fixation was applied using four pins: two inserted into the second metacarpal and two placed in the mid-distal radius (Figure 6). This setup was utilized to maintain ligament alignment and stability. 

**Figure 7:**
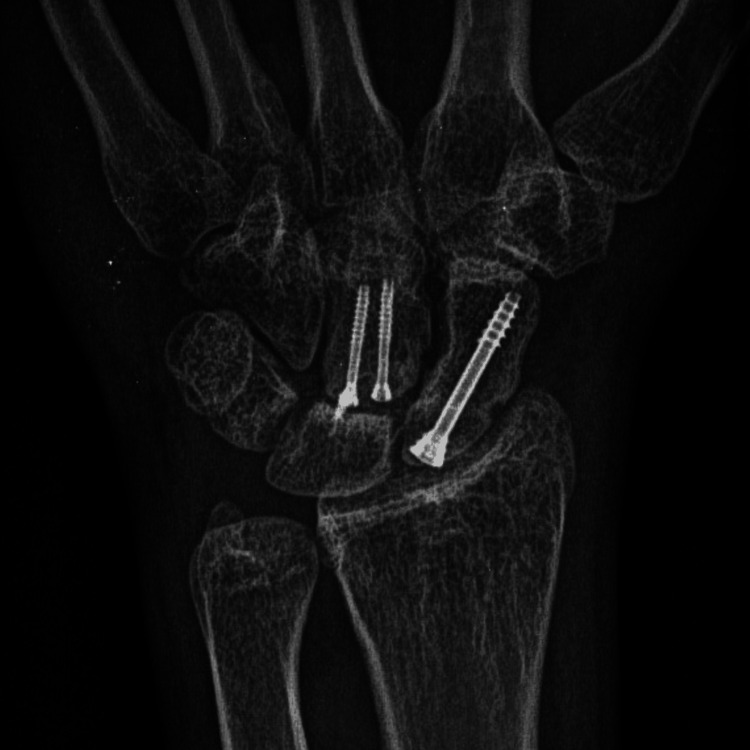
One-year follow-up X-ray showing the union of fractures.

**Figure 8:**
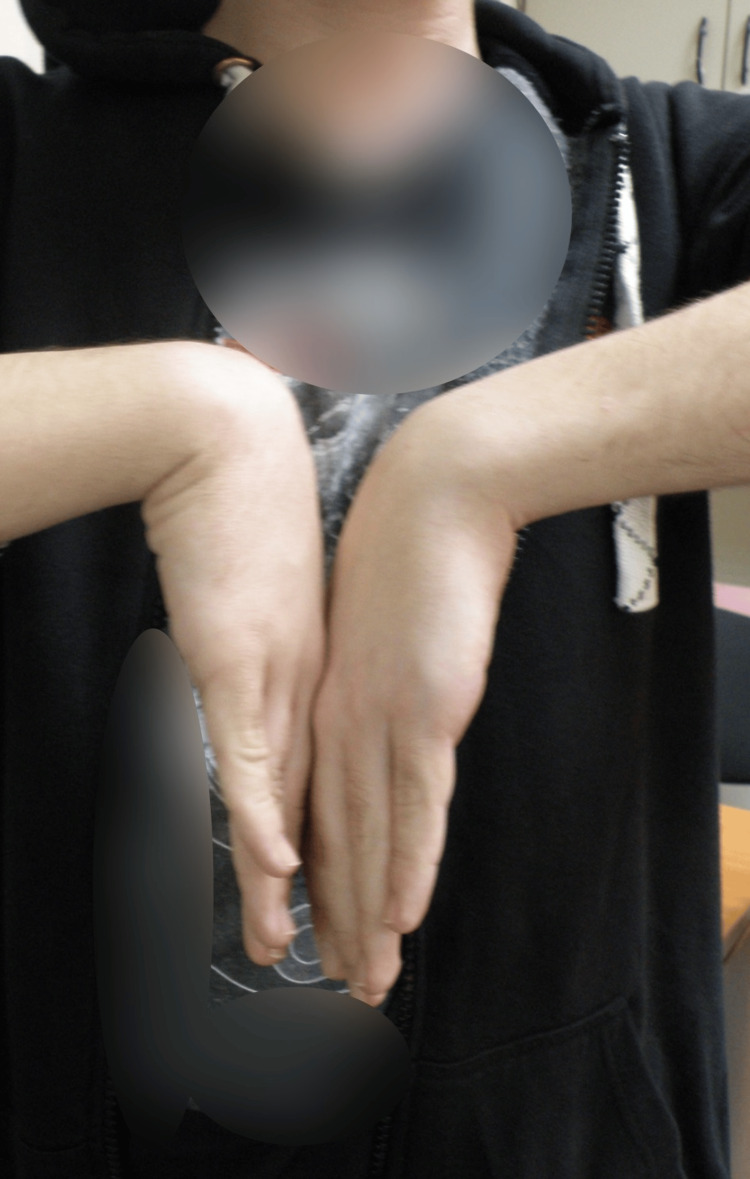
Clinical outcome of carpal flexion one year post-operative.

**Figure 9:**
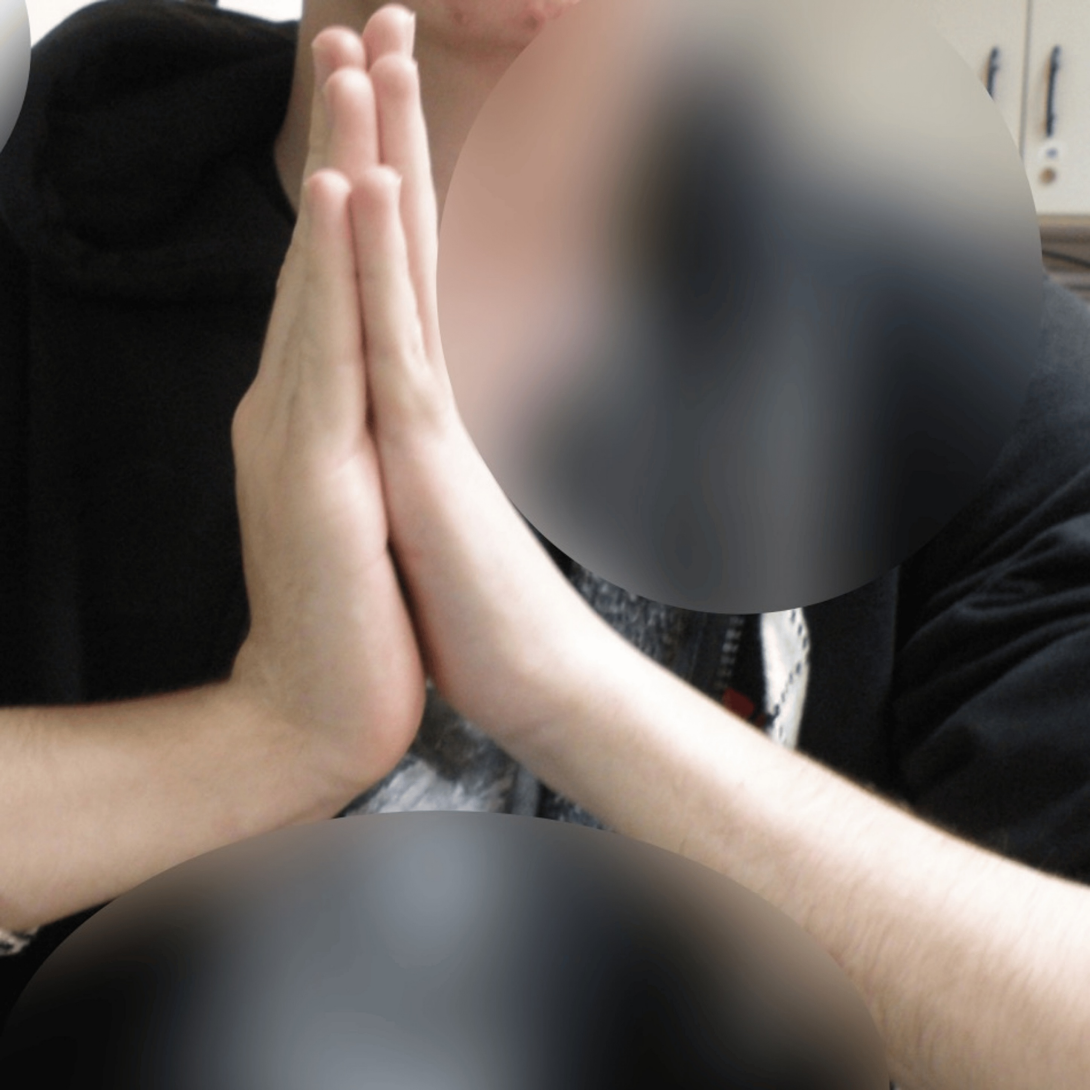
Clinical outcome of carpal extension one year post-operative.

**Figure 10:**
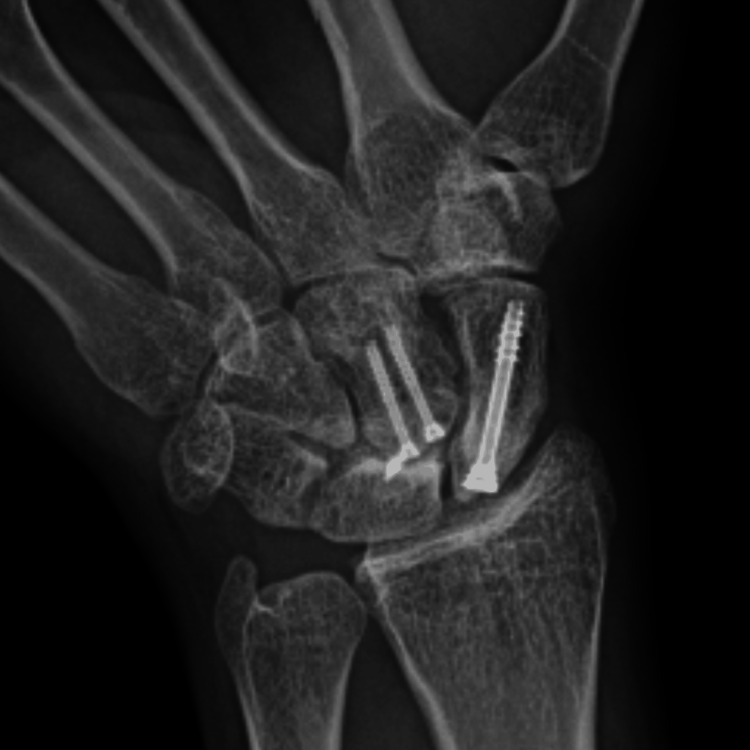
Five year follow-up X-ray showing no avascular necrosis of capitate and scaphoid bones.

**Figure 11:**
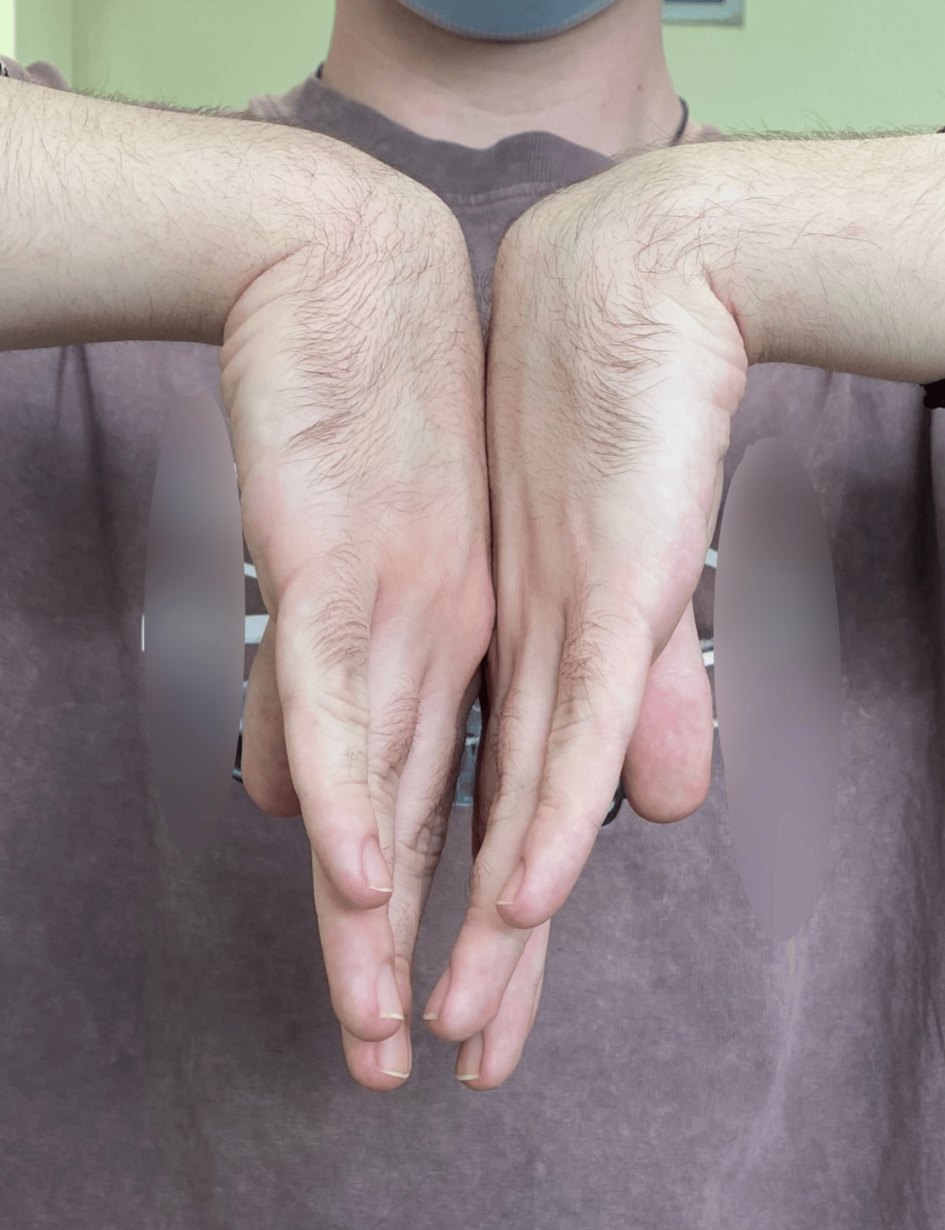
Clinical outcome of carpal flexion five years post-operative.

**Figure 12:**
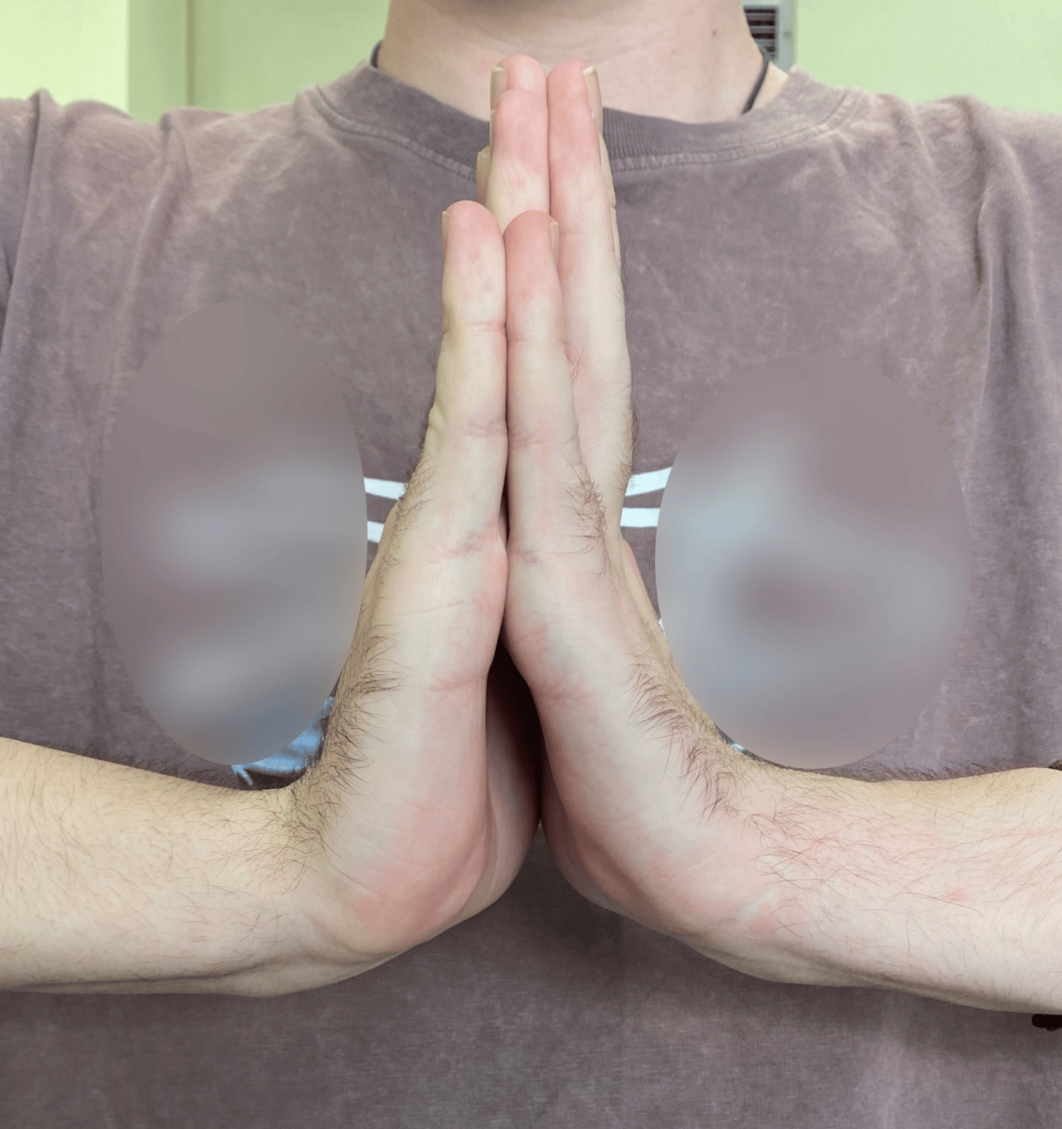
Clinical outcome of carpal extension five years post-operative.

In the postoperative course, the patient’s wrist was immobilized in a thumb spica cast. The external fixator and splint were eventually removed at 4 weeks, and mobilization, along with physiotherapy, was encouraged. At the one-year follow-up, radiographs demonstrated excellent fracture healing, with no evidence of arthritis and proper joint alignment (Figure 7). Clinically, the patient showed significant improvement, achieving a loss of only 15 degrees of palmar flexion (Figure 8). However, there was a residual 30-degree loss of dorsiflexion, although the patient reported no discomfort or pain (Figure 9). At the five-year follow-up, complete union of both the capitate and scaphoid fractures was confirmed, with no signs of avascular necrosis or arthritic changes in the midcarpal or radiocarpal joints (Figure 10). On physical examination, the patient exhibited a full range of motion in adduction, abduction, and palmar flexion of the wrist, with a 15-degree limitation in dorsiflexion (Figures 11, 12). The Modified Mayo Wrist Score was excellent, achieving 100 out of 100 points. 

## Discussion

Scaphocapitate syndrome is a rare entity in the hand surgery literature, particularly among adolescent cases, making it an infrequent yet intriguing diagnosis in this age group. A review of the literature revealed that the exact incidence of scaphocapitate syndrome remains unknown, but it accounts for approximately 0.6% of all capitate fractures [3]. As a result, many cases remain undiagnosed due to the rarity of the condition and the difficulty of detecting it on plain X-ray images, as the lesion may not be fully captured [2,6,8-10]. Okoro et al. highlight that X-rays have a limited diagnostic value in carpal fractures due to their moderate sensitivity and specificity, which can lead to missed fractures or misdiagnoses [8]. By contrast, CT scans, especially with thin slices and multiplayer reformations, provide near-complete accuracy when combined with clinical assessment. MRI further enhances diagnosis by underlining the presence of soft tissue injuries and occult fractures that may not be visible on X-rays.

The precise mechanism of injury leading to fractures of both the scaphoid and capitate bones, along with the 180-degree rotation of the capitate, remains a subject of ongoing investigation. Fenton initially described a sequence of events involving a fall on an outstretched hand with ulnar deviation. In this mechanism, the radial styloid fractures the scaphoid, transferring residual energy to the capitate, which subsequently also fractures [11]. This description fails to explain the 90- or 180-degree rotation of the capitate. Reviewing this, Stein and Monahan propose that an extended hand injury leads to a scaphoid fracture, permitting further hyperextension. This causes an immediate capitate fracture due to impact with the radius, accompanied by a 90-degree rotation of the proximal pole. As the hand returns to its neutral position, the proximal pole rotates an additional 90 degrees to realign with the carpus, ultimately resulting in a complete 180-degree rotation [12,13]. This proposed mechanism is consistent with our intraoperative findings. However, the delayed diagnosis often reported in the literature raises the risk of complications, including nonunion, avascular necrosis, post-traumatic carpal arthritis, and carpal collapse [2,5,6].

The literature suggests that open surgical fixation using either K-wires or screws is effective in achieving proper alignment, reduction, and fracture union. Regarding the treatment of scaphocapitate fractures, it is essential to restore the precise anatomical structure and achieve complete immobilization. This approach was successfully demonstrated by Dailiana et al. with two cases. In one case, the authors utilized two K-wires to secure the scaphoid fracture and one K-wire for the capitate fracture. In the second case, the scaphoid fracture was managed with a Herbert screw, highlighting the versatility of fixation methods in scaphocapitate fractures [2]. In our case, fixation of the capitate fracture with two Herbert screws and an additional Herbert screw for the scaphoid fracture aligns with the recommended guidelines to achieve stability and precise anatomical restoration. In most cases, this approach facilitates satisfactory fracture healing, allowing patients to resume their activities earlier [2,3].

In this case, the issue of delayed fixation, occurring three months post-injury, remains a point of consideration. Supporting the feasibility of delayed management, Kadar et al. reported a case of a neglected scaphocapitate fracture that was successfully treated with surgical fixation after a 30-year delay, with no evidence of avascular necrosis of the proximal capitate due to its adequate vascularity. They further emphasize that the use of cannulated screws is the preferred method for delayed fracture fixation, as it allows for optimal compression at the fracture site, thereby promoting successful healing [7]. This case underscores the importance of early advanced imaging in suspected carpal fractures and supports the viability of delayed surgical intervention under specific vascular conditions.

## Conclusions

Scaphocapitate syndrome is a rare injury pattern, particularly among adolescents, and is typically associated with high-energy trauma such as falls from height. Recognition of scaphocapitate syndrome in the emergency department can be challenging, as initial radiographic imaging is often inconclusive. Therefore, a history of significant upper limb trauma combined with the presence of effusion in the carpal region should heighten suspicion for this specific injury and other carpal bone injuries. Anatomical studies of the carpal bones associated with this syndrome suggest that delayed fixation of the fractures is a viable treatment option.

Recognition of the syndrome was challenging in the acute setting, as initial radiographs did not clearly demonstrate the full extent of the injury. However, the history of a fall from a significant height combined with clinical findings of carpal effusion prompted further investigation, ultimately confirming fractures of both the scaphoid and capitate bones. Although treatment was delayed, anatomical fixation ultimately led to excellent outcomes, with near-complete recovery of wrist function and a Modified Mayo Wrist Score of 100 at the five-year follow-up. This case underscores the rarity and diagnostic challenge of scaphocapitate syndrome in pediatric patients and demonstrates that, even with a three-month delay in treatment, meticulous anatomical reduction and stable fixation can restore carpal alignment and achieve favorable long-term functional and radiological outcomes.

## References

[1] Apergis E, Darmanis S, Kastanis G, Papanikolaou A (2001). Does the term scaphocapitate syndrome need to be revised? A report of 6 cases. J Hand Surg Br.

[2] Dailiana ZH, Papatheodorou LK, Malizos KN (2015). Scaphocapitate fracture: two cases with follow-up over 5 years. J Wrist Surg.

[3] Mortada H, AlKhudhair MR, Alsaygh EF, AlModumeegh AS, Kattan A (2022). A unique scaphocapitate fracture syndrome in an adolescent: a case report and a review of literature from the last decade. J Surg Case Rep.

[4] Hamdi MF (2012). The scaphocapitate fracture syndrome: report of a case and a review of the literature. Musculoskelet Surg.

[5] Yahyaoui M, Aharram S, Amghar J, Daoudi A, Agoumi O (2020). Carpal antelunar dislocation and Fenton syndrome: extremely rare association. J Clin Orthop Trauma.

[6] Pedrazzini A, Daci L, Bertoni N (2019). The scapho-capitate syndrome: a case report with follow-up of three years. Acta Biomed.

[7] Kadar A, Iordache SD (2023). Neglected scaphocapitate syndrome. J Wrist Surg.

[8] Okoro CK, Skalski MR, Patel DB, White EA, Matcuk GR Jr (2023). Imaging diagnosis and management of carpal trauma and instability-an illustrated guide. Life (Basel).

[9] Ameziane L, Marzouki A, Souhail SM, Daoudi A, Agoumi O (2003). Fenton's syndrome or scapho-capitate fracture (a case report) [Article in French]. Chir Main.

[10] Sawant M, Miller J (2000). Scaphocapitate syndrome in an adolescent. J Hand Surg Am.

[11] Fenton RL (1956). The naviculo-capitate fracture syndrome. J Bone Joint Surg Am.

[12] Stein F, Siegel MW (1969). Naviculocapitate fracture syndrome. A case report: new thoughts on the mechansim of injury. J Bone Joint Surg Am.

[13] Monahan PR, Galasko CS (1972). The scapho-capitate fracture syndrome. A mechanism of injury. J Bone Joint Surg Br.

